# Combining User-Centered Design and Lean Startup with Agile Software Development: A Case Study of Two Agile Teams

**DOI:** 10.1007/978-3-030-49392-9_3

**Published:** 2020-05-06

**Authors:** Ingrid Signoretti, Larissa Salerno, Sabrina Marczak, Ricardo Bastos

**Affiliations:** 6 University of Oslo, Oslo, Norway; 7 Monash University, Clayton, VIC Australia; 8 IT University of Copenhagen, Copenhagen, Denmark; 9 University of British Columbia, Vancouver, BC Canada; MunDDoS Research Group, School of Technology, PUCRS, Porto Alegre, RS Brazil

**Keywords:** User-Centered Design, Lean startup, Agile Development, Transformation, Case study

## Abstract

The combined use of User-Centered Design and Lean Startup with Agile Development has been pointed out by the literature as a manner to boost software development. User-Centered Design principles focus on providing tools for developers to better explore user needs and seek for a fitter solution. Lean Startup, on the other hand, supplements the triad combination by bringing the Build-Measure-Learn cycle and the concept of pivoting, either the problem understanding or the proposed solution. This paper reports on a case study of two software teams that have been undergoing the changes and impacts of such combined adoption. We investigated these teams for six months, from the moment that team members were trained on the job to grasp the essence of using the integrated approach inspired on Pivotal Labs proposal to the time they were considered mature enough to share their experiences with others within the organization. Through our in-depth study, we illustrate how this adoption promotes changes regarding to mindset, activities, practices, and techniques. We also report on the ‘team rhythm’ (or work flow) as experienced by the two teams. The paper contributes to current knowledge on the topic reporting on the changes and impacts that teams observed during the combined approach adoption.

## Introduction

Agile methods are defined by flexibility and adaptability in the context of building software products [3]. Despite the many benefits of adopting an agile method, the adoption still presents a lack of user involvement and participation [1], and product assertiveness. Vilkki [15] claims that agile must be combined with other approaches aiming to fill these gaps. Studies as Innodev [4], Converge [16], Nordstrom [7], and Lean UX [6] present models that combine agile with UCD and Lean Startup in order to boost the agile capacity in software development.

Using the combined approach requires a set of preconditions, especially when compared to using a single agile method. The studies report the need to define cross-functional teams, and the roles represent each methodology (e.g., software engineers - agile, product designers - UCD, and product managers - lean startup) [6]. Also, the adoption puts emphasis on focusing on identifying the problem to be solved rather than only worrying about identifying the scope [4].

Although the prerequisites mentioned in literature to adopting the combined approach, we still know little about what changes and impacts take place at the software team and that might be influenced by or depend upon the organizational level when facing the adoption. Motivated by the need to discuss the modifications inherent to the adoption process, we conducted a case study with two software teams from a large-scale company. Our research reports from a team perspective the changes related to the teams’ mindset, activities, practices, techniques, and rhythm to accommodate the combined approach adoption. Our main contribution is providing an understanding of how the combined use adoption promotes several impacts on the team’s software development process. The findings offer inputs to the academia and industry practitioner.

The reminder of this paper is organized as follows: Sect. 2 details the research method. Section 3 reports on the main results. Section 4 discusses the main findings and explores the paper contribution. Section 5 presents previous studies and a comparison with our results. Section 6 concludes the paper with our study limitations and proposals of future work.

## Research Method

We conducted a case study [12] with two teams from a multinational company named ORG (name omitted for confidentiality reasons). Next, we introduce the case setting and the data collection and analysis methods.

### Case Setting

We aim to present the changes and impacts perceived by two software teams in an adoption process of a combined approach composed of Agile, UCD, and Lean Startup. Therefore, we briefly explain the case setting, including the company’s previous scenario aiming to emphasize and to contextualize the modifications. We also present the product scope each team is responsible for.

*The Company.* ORG has development sites in the USA (headquarters), India, and Brazil. With over 7,000 employees and responsible for about 1,200 internal software products, the IT department started its agile transformation in 2015 and moved to the combined use of Agile, UCD, and Lean Startup principles in late 2017. Before adopting the combined approach, ORG had a well defined roadmap for software product improvements based on an annual budget negotiated among business department and organized into software projects. High-level business features were prioritized and decided upon business personnel to later be transformed into software requirements by IT software project teams. The project deadlines were strict and defined by quarter, i.e., every four months the project teams delivered a set of software features to existing or new software products to the company internal customers.

Associated with the business features definition negotiation, the company had Business Representatives responsible for defining the business needs. Once approved those needs were translated into business features, elected as the starting point for the IT project teams. Mostly, IT Business Analysts transformed these features into software requirements with the help of the Business Representatives and used these to drive software development.

With the introduction of the agile transformation in 2015, project teams used Scrum as the guiding development framework. From this time and on, it become common but not company-wide spread to get more team members (e.g., developers, software architects, testers) engaged into the business feature-to-software requirement translation. Some teams move then to a more product-oriented view while others are still guided by project time slots. The company starts then to discuss how to move from a world-wide roadmap to a product development organization when they realize help was need. This is when they decide to board the agile, UCD and Lean Startup combined journey and hire Pivotal consulting to support such transformation.

Overall, Pivotal brings the Pivotal Labs^1^ methodology at core of the transformation. This methodology proposes a ‘team rhythm’ (or work flow) composed of principles and ceremonies based on the three before-mentioned approaches. It also suggests the adoption of a cross-functional team composed of three leading roles: Product Designer, Product Manager, and Software Engineer. The Pivotal Labs’ main goal is to help teams to build software products that deliver meaningful value for users and their businesses. Thus, it offers a framework and initial starting point for any team to discuss the client/user specific needs and define its way towards software development.

The transformation and adoption process is the subject of interest of this research. In order to understand the process, we conducted the study with two software teams that were already half-way to the understanding of how to become product software teams. We present the teams’ background next.

*Teams’ Context.* We observed *in-loco*, in a lab at the University campus, two teams from the financial area located in Brazil. The lab was intentionally prepared for the teams to work on as part of a PUCRS and ORG research agreement. Both teams develop software product for the company internal use. The teams are composed of 2 Product Managers, 1 Product Designer, and 4 Software Engineers each. Team A is responsible for a software product that calculates the associated cost services offered by the products sold by ORG and displays this information to ORG consumers. The software consolidates information about services offered by the company, such as sale, installation, and equipment configuration, and stores employee data and hours spent on the provided services. Data are consolidated into a projects by served customer for another product team from the financial area to use this information as input for their product use. Team B is responsible for the software product that gathers information about these services generated by ORG software products and stores them for Team A to use. The team has the goal to automate the calculation average of the equipment and services costs offered by the Brazilian site. Sequentially, the application performs the analysis of these multiple data aiming to provide consolidated information to the accounting area, which uses these data for internal control and reports for the company. Table 1 presents the participants’ profile per team.

**Table 1.** Participants’ profile

Team	Role	IT work exp	Company exp
Team A	Product Manager	21	6
Team A	Product Manager	16	7,5
Team A	Product Designer	27	10
Team A	Software Engineer	6	1
Team A	Software Engineer	21	8
Team A	Software Engineer	5,5	4
Team A	Software Engineer	20	11
Team B	Product Manager	19	0,5
Team B	Product Manager	23	10,5
Team B	Product Designer	5	4
Team B	Software Engineer	10	4
Team B	Software Engineer	15	11
Team B	Software Engineer	7	7
Team B	Software Engineer	5	5
–	BR Transformation Lead	12	7

### Data Collection

We observed the two product teams for a 6 months-period and we used multiple data sources to conduct the study. Following, we present each data collection method and its related purpose within our study.

*Questionnaire.* It was used to collect the participants’ profile (name, role, main responsibilities, time in years working in IT and at ORG, and whether the person participated of the immersion training in the US.

*Semi-structured Interviews.* They were used first to gather information on the team members perceptions about the combined transformation, the training experience, and benefits and challenges. This interview was extended to the Brazilian Transformation Leader. A second round of interviews were conducted with all team members to gather their perception on team roles changes, interaction among roles, and impact of changes on the work routine. Interviews were also generally used as a means to follow-up and learn more details about diverse aspects unveiled in the observation sessions. All interviews were voice recorded and transcribed for analysis. None lasted more than 30 min as previously agreed with the industry research project sponsor.

*Daily Observations.* These were conducted to observe team ceremonies (e.g., daily standup, retrospective, iteration planning), meetings with stakeholders (user interviews, demos), and work routine. We also conducted shadowing of roles (e.g., product manager, product designer, and software engineer) seeking in-depth knowledge about the responsibilities of each role.

*Focus Group.* We performed six sessions, 3 of them were overall follow-ups and confirmation of data collected through other methods (e.g., to discuss in-depth the Product Designer new role). Moreover, specifically, one session was conducted aiming to consolidate the teams perceptions about the benefits and challenges of the combined approach (reported in [14]). Another session focused on the discussion of the elements of each approach as perceived by the teams (e.g., activities, techniques, and work products). And another session aimed to confirm the mapped elements of each methodology (e.g., naming, meaning, context of use, etc as observed in their daily work routine) into the combined approach representing the team work flow (or team rhythm as called by Pivotal Labs). In this last session we also asked the participants to visually represent this work flow as they saw fit. Each focus group session lasted in average 1.5 h, except the last one that lasted about 3 h (previously arranged with the teams). All sessions were voice recorded and transcribed.

**Fig. 1.**
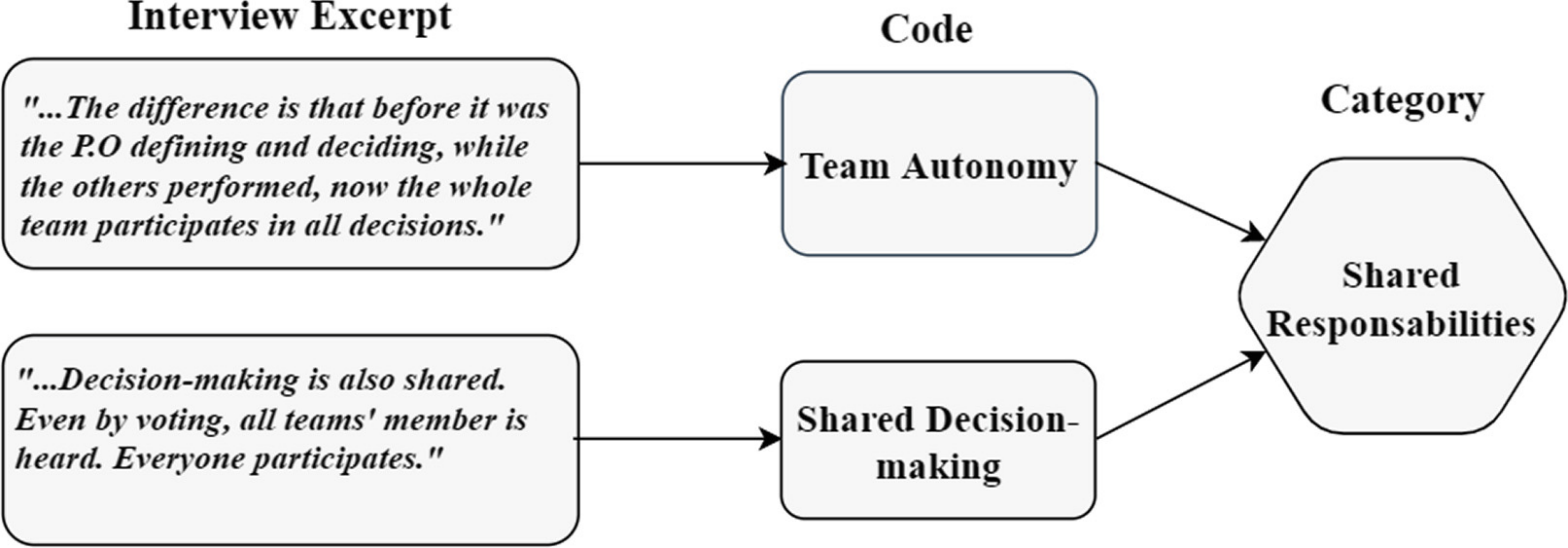
Code analysis example

### Data Analysis

Regarding data analysis, we conducted the content analysis procedure by Krippendorff [9], using a qualitative approach to the ethnographic content analysis, where we are focused on the narrative description of the situations, settings, as well as the perspective by the actors involved in the phenomena. Also, as we use recording/coding units, we organized the analysis into the following steps: organization and pre-analysis, reading and categorization, and recording the results and using Atlas.TI^2^ tool. We first read the dataset, extracted text excerpts, and marked them as codes (see an example in Fig. 1). These codes were revisited and grouped into larger codes, forming categories. We constantly reviewed our coding scheme with the two seniors researchers (the last two authors) aiming to mitigate any limitation or bias in our analysis. The two senior researchers also reviewed the questionnaire and interview scripts and supported the piloting of these instruments for face and content validity with an invited researcher with previous experience working with agile teams in industry.

## Results

The case study results reveal aspects related to the combined approach adoption and usage. For instance, the *product is developed under a new perspective*, using a problem-oriented mindset which included the teams’ changes to working attitudes to adapt to this new mindset (Sect. 3.1). We also highlight changes related to *methodological aspects* (Sect. 3.2), such as the addition of UCD activities to promote user involvement and participation. Also, the use of the Build-Measure-Learn loop guided by the underlying concept of experimentation from Lean Startup as a means seek for the proper product solution. Or yet, changes to the current already adopted XP practices to improve quality of code and constant releases. We describe these and other relevant results next.

### Product Developing Under a New Perspective

In our previous study [14], we presented the company decision for migrating from agile to the combined approach, including the transformation package of activities to train people. Here, we discuss changes in the teams’ day-to-day work, including those that reflect upon or depend on organizational decisions. We start by presenting the change from a project-base structure to a problem-based mindset-oriented way of working.

**Problem-Oriented Mindset.** Teams’ members mentioned that one of the most relevant changes experienced during the transition was moving to a problem-oriented perspective to seek for the user needs understanding rather than refining software requirements only:

*“Before, we usually received a set of predefined requirements. We implemented these requirements and considered our work done. We did not know whether the problem was solved or not. Now, we do participate in and have the opportunity to investigate and understand the problem.”*

      Team A experience

The participants also considered that the change in mentality was a challenge at first, as this modification directly affects the team’s attitudes. The mindset change required that team members start acting as main actors in the development of the product and not just as those who operationalize it. However, it is crucial an ownership attitude from the teams to fit in this new mentality.

**Team Engagement.** The teams’ commitment to the entire software development process has increased considerably since the adoption of the problem-oriented mindset. In fact, the teams started to recognize the need to move to an improved way to provide more business-aligned products, changing at the core the manner of understanding the product, during the hands-on training on the new combined approach. This realization led them to understand that achievements were dependent on the team involvement with changes. For instance, they promoted a shared product vision:

*“Everyone needs to understand the product, not just the product designer or the product manager - the software engineer is no longer isolated. The entire team needs to know why the products are working and have an understanding of the product vision. Everyone is always up-to-date.”*

      Team B experience

**Shared Responsibilities.** With a shared product vision is essential that teams have shared responsibilities. The whole team participates from activities as the problem understanding - where is discussed the product’s needs. By establishing a relationship between them and the stakeholders, the team can define a stakeholder map - which allows the teams to be more effective in the next phases of the product development, as well. This change requires a different position from the software engineers since the product designer and product manager already have this participative role with the stakeholders due to the nature of the roles. Now, the software engineers affirm that they need to adapt to a more collaborative attitude in all decisions that involve the product:

*“We have the responsibility to guarantee the environment to the solution developing, make the pipeline implementation using continuous delivery and integration. However, we are now responsible for participating in each decision in the team since the conception of the product, joining the users’ interviews, stakeholders meeting, and the other ceremonies.”*

      Software Engineers from team A and B experience

In the teams’ perspective, in terms of methodological aspects, the combined approach adoption depends strongly on the first two elements discussed above. Having established that, we can describe the aspects related to the teams’ way of working on the adoption of UCD and Lean Startup concepts, also the change from the Scrum framework to the XP methodology.

### Methodological Aspects

**Fig. 2.**
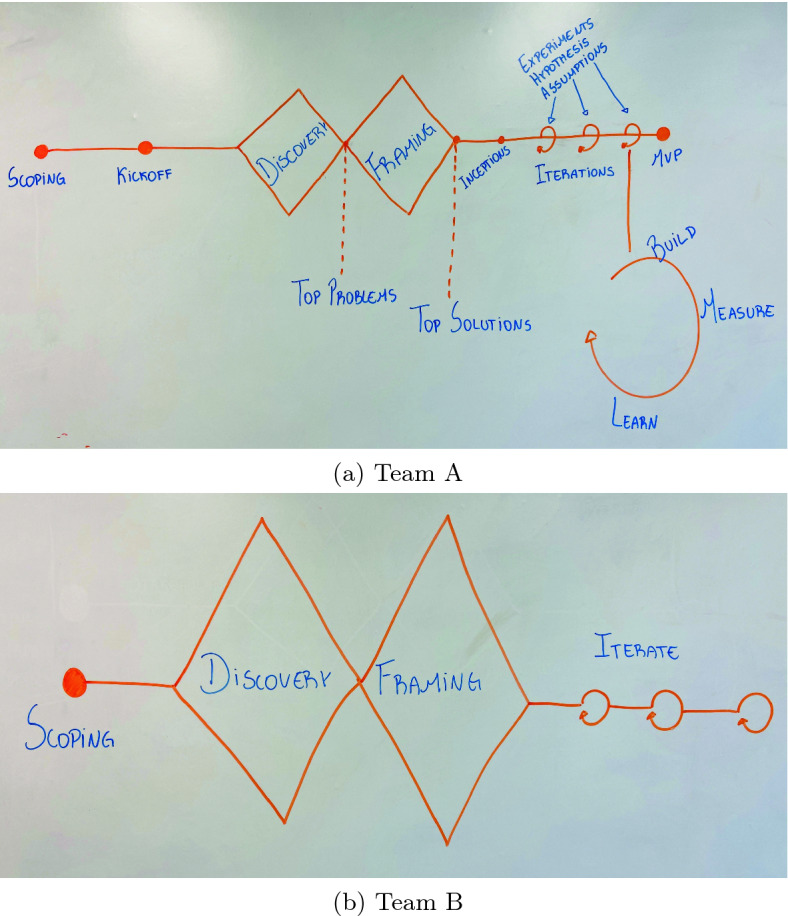
Team A and B work flow (or team rhythm)

As previously mentioned, we asked the teams to visually illustrate how they perceived the changes related to methodological aspects that guide their work. Figure 2 shows the teams original representations.

Following the teams’ representation, next, we start exploring the aspects related to UCD as a manner to promote a user involvement in the product development in the teams’ perspective.

**UCD to Promote User Involvement and Participation.** Although agile methods encourage the relationship between team and stakeholders, the participants felt that in their context, it seems to be not enough because the product still not address the stakeholders’ needs. With the change for the combine approach usage, business people and users reported a set of benefits in terms of user participation in the teams’ activities and their daily work, and also the user involvement, since with the introduction of UCD activities, techniques and concepts they seem to be more engaged in the product development.

The participants considered as a significant modification in the UCD context the adoption of the Discovery and Framing framework designed by Design Council^3^. On the two teams’ representation (Fig. 2), the framework is in the early stages, aiming to explore the problem and possible solutions to it.

Team members emphasize that the use of discovery and framing framework, it is a consequence of work in a problem-oriented, reaffirming once again the mindset change impacts. Another relevant factor for participants is the need to have the whole team working in the framework stages, as problem exploration, user interviews, user research, and other activities. Team A members consider that team engagement to participate in these activities promotes an approximation with the stakeholders, making them believe in teams’ effectiveness:

*“We gain their (stakeholders) trust when we talk with them and show interest in providing a product that attends and solves their problems.”*

      Team A experience

Team B participants declare:

*“Using UCD techniques and also a mindset of being more empathetic with our stakeholders, make them feels indispensable in the development process, and consequently, encouraged to talk and to contribute with us. Our stakeholders see us as problem solvers. We gain their trust when we show interest in providing a product that attends the problems.”*

      Team B experience

The problem understanding is an outcome of the problem exploration. This outcome allows possible framing solutions to the given problem. The teams reported that the stakeholders’ presence is even more necessary at this stage. Team A members affirm that to promotes the stakeholders’ engagement is vital to collect stakeholders’ feedback all the time and consider it:

*“We use stakeholder feedback as a tool to refine and redefine problem definition and priority. Being aligned with the stakeholders’ needs makes them more confident about our work. We work together with stakeholders, ensure that the developed product is being following the right path.”*

      Team A experience

In team B experience, another concept that helps to focus on the problem understanding and provide a more accurate solution to the product is experimentation concept inside the problem discovering:

*“We aggregate value to our products by using experimentation. We explore the problem that business brings to us, and by the end of it, we address their needs in the product.”*

      Team B experience

Also, addressing the users and business needs in the product, demand a change of mindset to guide the teams. They mentioned that an important value taken for all three methodologies is the BML loop and experiments, which lead us to explore the teams’ perspective on the lean startup concepts addition.

**Lean Startup Concepts as a Tool to Be More Assertive.** One of the most powerful concepts derived from Lean Startup in the teams’ point of view is the BML loop inclusion. The participants have defined the BML usage as an approach, and the reason for that is that loop is applied all the time:

*“We use BML all the time in any part of our process. For example, a user interview. If we are defining the interview script, we are building the script. We measure the script value by observing after the interview, if we collected the right data or not (e.g., the stakeholders answer the question, but we do not formulate the question for the answer that we aimed.) - and this process allows us to learn from our fails to create a more assertive script to be more accurate in the next one. BML is applicable to any product development activity.”*

      Team A experience

However, BML usage is not so relevant if used alone. The richness of the loop is combined with experimentation, as teams’ members reported:

*“All foundation of the BML brings the experimentation concept in the core of it. We work with a problem-oriented mindset because the experimentation allows it. In the beginning, we have a simple problem view, and this leads us to start making assumptions from that, execute the experiments using prototypes or any technique. The results give us a condition to measure it and to refute or accept our assumptions. At the end of it, we learn from the results and restart the loop, refining our vision.”*

      Team A and B experience

Team A also experienced an unusual usage of experimentation in a non-software solution. They mentioned that this shows the relevance of the concept usage for the teams, stakeholders, and to the company itself:

*“Our users were claiming a solution to the performance issue in the system. Before we run directly to the code, imagining that the problem in the software solution, we decide to analyze the problem. The stakeholders reported that the use of some spreadsheets contained a significant amount of data, and it was getting a poor performance taking about three days to calculate and return the results. So, we assume that maybe the problem was not in the application, but in the host machines. We decided to run the same application in a more powerful machine, and we have found out that our assumption was right - the problem was in the machine’s performance. This experience shows us the relevance of experimentation - and more than that, it shows that sometimes the problem solution could not be a software solution, which for us is a huge breakthrough.”*

Team A experience

Besides, the participants perspective, experimentation gives them room to fail up; however, fail and fix quickly:

*“Product development is uncertain and very susceptible to failure. Nevertheless, what matters is the speed at which we will react to those. The experimentation as a core of the BML gives us room to fail but also allows us to fail and fix quickly. We do not need to wait until the end of the iteration to discover that we do not understand the stakeholder needs.”*

      Team A and B experience

Related to the pivot/persevere usage, the concept follow the same idea of one of the agile principles, in terms of adaptability for team B members. They affirmed that pivot/persevere reinforce the relevance of refining the product and problem strategy, being adaptable to change or persevere:

*“Experiments give us conditions to understand if we are in a smart strategy for our product or not. Also, the stakeholders’ relationship with us is an essential factor to persevere in the strategy or start to look another direction, pivoting. Sometimes, the strategy defined in the long-term can not be valid anymore. That is the reason why BML, experimentation, and pivot/persevere perform better together; one depends on the other.”*

      Team B experience

Notwithstanding, the addition of UCD and Lean Startup has been the main change. In terms of code development, the teams reported a need to align the changes in a possible technological manner. To attend this modification, participants reported the use of XP instead of Scrum as an agile method. Now, we explore how the insertion of XP affected the process, from the teams’ perspective.

**XP to Boost Code Quality.** The XP methodology choice as an agile method came with the Pivotal Labs approach proposal. However, team A members recognize that even that the change was top-down from Scrum framework to XP was a great fit. They cited that the use of XP practices (e.g., pair programming, TDD, and unit test) boost the development and increase the code quality:

*“The use of pair programming increases our product development process. We can benefit from using it in many ways: from accelerating the learning process of a new engineer, to promote improvements in the code quality.”*

      Team B experience

Continuous Integration (CI)/Continuous Delivery (CD) pipeline was considered as a practice that promotes a problem-oriented mindset in the context of software development, as team B participants mentioned:

*“CI/CD pipeline was crucial to address the changes. It promotes faster feedback and help us to validate stories on the production environment. CI/CD inclusion encourages software engineers to feel more proficient.”*

      Team B experience

The participants also reported significant modifications in terms of the team rhythm. They have changed a set of ceremonies during the daily and the iteration work and also its nomenclature aiming to attend to XP methodology rhythm:

*“We tried to be more aligned, and the ceremonies are useful for that. We continued doing the standup meeting, retrospective, and planning. However, we now have an office standup to be more connected to other teams - also, the ceremony nomenclature change from sprint to iteration. In the planning sessions, we choose if we must have more than one session, for example, a pre-iteration meeting. Finally, we have weekly sessions with all stakeholders to strengthen our relationship with them further.”*

      Team A and B experience

Once again, BML shows its relevance, as well as experiment concepts in teams’ perspectives. The teams reported that the use of these concepts impact the manner they deal with the iteration directly. It is a common-sense between them, the relevance of developing the product, thinking more systematically and investigating the real problem, defining assumptions, executing the experiments, collecting data, and verifying whether the assumptions were accepted or refuted.

Concluding the teams report, the participants attributes the adoption success in terms of mentality, engagement, and modifications related to methodological aspects with UCD and Lean Startup, to a organically approach application:

*“Even though our drawing represents a sequential or continued vision of the methodologies combination, our daily use is adapted. If we are during the iterations and perceived that the problem is not well defined, we are ok to come back to the discovery and framing framework and start again. Alternatively, if we defined some assumptions and discovered that the product/problem vision is not aligned, we can redefine these assumptions. This is secret of the adoption, apply the approach organically.”*

      Team A experience

## Discussion

Schön et al. [13] mentioned the barriers of access the stakeholders as a challenge in their study. In the reported study, mitigate this barrier was considered as one of the crucial changes that derive the way that the company works now; it is working as a problem-oriented perspective. The teams changed their mindset to map the user and business problems over only refine pre-defined requirements - solving the difficulty of decrease the creativity to the process of solution-finding.

Teams’ attitude required an adaption to attend the problem-oriented mindset change. All roles became more engaged in activities as product/problem scoping, user interviews, or stakeholder meetings. Nyfjord and Kajko-Mattsson [10] mentioned in their study that the entire team engagement in these activities often was executed by business people and the teams (especially software engineers) only receive the artifact produced from these activities. Once again, these problems are decreased by changing for the problem-oriented mindset.

Reinforcing the development-oriented by user/business problem perspective, there is an extensive effort on the discovery of the right problem and framing the possible solutions to the right solution. The double diamond structure that the teams applied follows the UCD activities defined at ISO 9241-210 [8]. Schön et al. [13] also defines that this is one of the critical aspects under the integration of UCD and Agile, separate product discovery and product solution. Define the discovery and framing usage brings benefits associated with the added value of the product. Alahyari et al. [2] mentioned that one of the factors that can impact the perceived value on the products is the customer relationship, which is highly explored during the discovery and framing since the UCD activities and techniques usage promotes an approximation between team and stakeholders.

Incorporated to the discovery and framing and also in the iteration, the teams make use of the build-measure-learn loop, aiming to produce a better product. The perceived benefits and the reason for the teams choose to use build-measure-learn derived by experimentation was very similar to those reported by Yaman et al. [17], which reduce the development effort, deeper customer insights, and use experimentation as a guide on development decisions. The teams also reported that the use of a build-measure-learn application was a considerable modification since they work only with agile methodologies before, and they feel that agile does not help them to know what product should be developed. Edison, Wang, and Abrahamsson [5] affirm the same, agile prescribes how to develop, but it is not so accurate to answer and to investigate the products’ needs.

Another finding on the combined approach adoption is the use of the pivot and persevere concept original from lean startup [11]. Pivot decision could occur at any moment (e.g., problem/solution definition, scope definition), as well as remain in the same strategy, persevering. This is relevant because inputs to the teams and does not allow the teams to work on products that will not add value to the customers and business people, reducing the waste of the process [11].

The change impacts, related to the insertion of XP practices, were lower since the teams were already familiar with agile methods. However, the change for an XP over scrum framework affects their way of work. The inclusion of the build-measure-learn loop and also the XP practices as pair programming, TDD, and continuous delivery bring perceived benefits to the teams and stakeholders.

As reported, the manner of how the combined approach is adopted is essential. It is possible to notice that even that concepts from UCD and Lean Startup are essential in their new way of work, the core of the approach remains in agile value, which is a response to change over following a plan [3], which means use the approach adaptively. Pivot/persevere concepts explore in the core of it, the change of the team rhythm adopting XP ceremonies, which was claimed to promote the engagement and involvement among the team members and stakeholders. From a team’s perspective, these modifications ensure adoption success.

## Related Work

Combine UCD and Lean Startup with Agile software development have been a hot topic in the context of software development [4, 16]. In this section, we aim to compare our findings in light of the literature findings of the subject.

Lean UX [6] philosophy is grounded on Agile software development, Design Thinking (DT), and Lean Startup methodologies. This philosophy has focused on the design process incorporated into the development of a product that had defined principles based on the concepts of the three methodologies (e.g., cross-functional teams from DT, permission to fail from Lean Startup, and getting out of the deliverable business from agile). Although the principles are related to the combined approach presented in this study, the fact of Lean UX’s focus on the design process illustrates the difference from our case study, which explores the combined approach adoption in the software development context. Nordstrom [7], Converge [16], and InnoDev [4] models also proposes a combined approach of Agile software development, Design Thinking (DT), and Lean Startup. However, the models are focused on software product development. In Nordstrom and converge (which was inspired by Nordstrom), starts applying DT, right after Lean Startup concepts BML, experiments, and pivot and persevere, in the end, the sprints are guided by BML concept also. InnoDev model, on the other hand, starts with an initial phase of scoping, which uses elements from DT, and follows the same flow used by the other two models above.

Similarities could be observed from the literature studies, and our case reported. The double diamond usage and the concepts as BML and experimentation are present Nordstrom Model. Also, the models propose through a set of techniques derived from DT and Lean Startup, the problem-oriented mindset.

However, compared to our study, the literature findings have aimed to propose models for the combined approach. Our studies does not proposes a model. We aim to reports by agile teams’ perspective from a multinational company, how UCD, Lean Startup, and Agile are adopted and used in their daily work. Nordstrom and Converge models were evaluated in startups, and even though InnoDev was designed for small to large companies, it was not evaluated empirically. Also, this difference implies that these studies do not have the whole context of persuading users and business people to believe in the adoption.

Another difference compared to our case study and literature findings is the use of UCD over DT. Moreover, also, BML usage is applied from the middle to the end of the presented models. In our study, the teams reported the use of BML during the entire process, followed by experiments. Finally, the models propose the use of the Scrum framework just using some XP practices - in our case study, the teams fully adopted XP practices, techniques, and rhythm (ceremonies).

The comparison between our case study and literature findings gives an understanding of the need for a detailed characterization of the combined approach by teams’ perspective, which were the most affected in the adoption. This richness of detail was observed in none of the studies. Also, reinforce the relevance to recognize how this kind of transformation takes place in a large-scale setup.

## Conclusion, Limitations, and Future Work

We reported through a case study the perspective of two teams about the combined approach adoption composed of UCD, Lean Startup, and Agile Software Development. The detailed characterization provided in this study reveals that the adoption is comprised of a set of elements as a new problem-oriented mindset, team engagement, and these two above provides methodological aspects changes.

Also, it is relevant to affirm that UCD and Lean Startup in software development were a significant finding from the study results. UCD contributes by promoting user involvement and Lean Startup with BML usage as an approach, having experimentation in the core. An important conclusion, this combination has the concern of stays adaptable and its usage in a more organic way are characteristics of agile methods that remain at the core of the combined approach.

For the academy audience, our study contributes to essential details about the elements and essence that surrounds this approach. The industry practitioners will take advantage of the described study used by a multinational company and how this approach fits in the software development process setting.

Inherent to any empirical study, this study has limitations. Construct validity regards whether the scenario of study is representative of the real world while external validity is concerned with generalization. We observed two teams in a real setting, which offers them a new setup that aims to promote collaboration. Also, the teams are composed of members playing distinct roles and with different experiences. Moreover, we used interchangeably and overtime multiple data sources aiming to triangulate our findings, which were reviewed continuously by senior researchers. Therefore, although we cannot claim that our results apply to distinct scenarios, these strategies helped reduce limitations.

As future work, we suggest, the replication of the study in other companies with the same configuration, aiming to compare the findings; also, another valuable work could be compare teams who adopt the combined approach and those that use another approach (e.g., Scrum, Kanban), aiming to discover the strengths and weakness of the approach compared to other agile methods.

## Footnotes

1 https://pivotal.io/Labs.

2 atlasti.com.

3 https://designcouncil.org.uk/news-opinion/what-framework-innovation-design-councils-evolved-double-diamond.

## References

[1] Abelein U, Sharp H, Paech B (2013). Does involving users in software development really influence system success?. IEEE Softw..

[2] Alahyari H, Svensson RB, Gorschek T (2017). A study of value in agile software development organizations. J. Syst. Softw..

[3] Beck, K., et al.: Manifesto for agile software development (2001). https://www.agilemanifesto.org/

[4] Dobrigkeit, F., de Paula, D.: The best of three worlds-the creation of INNODEV, a software development approach that integrates design thinking, SCRUM and lean startup. In: Proceedings of the 21st International Conference on Engineering Design, pp. 319–328. Design Society (2017)

[5] Edison, H., Wang, X., Abrahamsson, P.: Lean startup: why large software companies should care. In: Proceedings of the International Conference on Agile Software Development, pp. 1–7. ACM (2015)

[6] Gothelf J (2013). Lean UX: Applying Lean Principles to Improve User Experience.

[7] Grossman-Kahn, B., Rosensweig, R.: Skip the silver bullet: driving innovation through small bets and diverse practices. In: Leading Through Design, pp. 815–830 (2012)

[8] ISO: 9241-210: Ergonomics of human system interaction-Part 210: HCD for interactive systems (2010). https://www.iso.org/standard/52075.html

[9] Krippendorff K (2018). Content Analysis: An Introduction to Its Methodology.

[10] Nyfjord J, Kajko-Mattsson M, Wang Q, Pfahl D, Raffo DM (2008). Degree of agility in pre-implementation process phases. Making Globally Distributed Software Development a Success Story.

[11] Ries E (2011). The Lean Startup: How Today’s Entrepreneurs Use Continuous Innovation to Create Radically Successful Businesses.

[12] Runeson P, Höst M (2009). Guidelines for conducting and reporting case study research in software engineering. Empirical Softw. Eng..

[13] Schön E-M, Winter D, Escalona MJ, Thomaschewski J, Baumeister H, Lichter H, Riebisch M (2017). Key challenges in agile requirements engineering. Agile Processes in Software Engineering and Extreme Programming.

[14] Signoretti, I., et al.: Boosting agile by using user-centered design and lean startup: a case study of the adoption of the combined approach in software development. In: Proceedings of the International Symposium on Empirical Software Engineering and Measurement, pp. 1–6. IEEE (2019)

[15] Vilkki K, Abrahamsson P, Oza N (2010). When agile is not enough. Lean Enterprise Software and Systems.

[16] Ximenes BH, Alves IN, Araújo CC, Marcus A (2015). Software project management combining agile, lean startup and design thinking. Design, User Experience, and Usability: Design Discourse.

[17] Yaman S (2017). Introducing continuous experimentation in large software-intensive product and service organisations. J. Syst. Softw..

